# Water Sorption and Mechanical Properties of Cellulosic Derivative Fibers

**DOI:** 10.3390/polym14142836

**Published:** 2022-07-12

**Authors:** Mathilde Simon, René Fulchiron, Fabrice Gouanvé

**Affiliations:** Univ Lyon, CNRS, UMR 5223, Ingénierie des Matériaux Polymères, Université Claude Bernard Lyon 1, INSALyon, Université Jean Monnet, CEDEX, F-69622 Villeurbanne, France; mathildejoyce.simon@gmail.com (M.S.); rene.fulchiron@univ-lyon1.fr (R.F.)

**Keywords:** water vapor sorption, cellulosic fiber, Park model, tensile properties

## Abstract

In this study, water vapor sorption, desorption properties and tensile mechanical properties of four cellulosic fibers, cotton (C), flax (F), viscose (V) and cellulose acetate (CA), were determined. The sorption and desorption isotherms were modeled using the Park model, which allowed an accurate fitting on the whole range of water activity. This model corresponds to a multi-sorption mode dividing in three sorption modes: Langmuir sorption, Henry’s law and water clustering. Park’s parameters were compared for the sorption and desorption isotherms for each fiber. Regardless of the fiber, differences between sorption and desorption were obtained only for the Henry sorption. The obtained sorption properties were correlated to the accessibility and the amount of sorption sites and also to the crystallinity level of the fibers. It was found that V exhibited the highest water sorption capacity due to a higher hydroxyl groups accessibility and a low amorphous fraction, followed by F, C and CA. Results from tensile tests demonstrated that F and C fibers were more rigid, more resistant and less ductile than CA and V fibers due to a difference of microstructure of the fibers. Finally, the presence of water-sorbed molecules led to a decrease in tensile modulus due to plasticization phenomenon.

## 1. Introduction

Cellulosic fibers have been used as structural materials since prehistoric times. More recently, interest in the use of materials derived from natural resources has increased dramatically. Environmental concerns such as global warming, energy consumption and the desire to obtain products from renewable sources have led to a resurgence of interest in plant-derived products. Plant fibers are very attractive and are used for a wide variety of industrial applications such as textile for fabric making [1], automobile and building industries as reinforcement in composites materials [2]. They are cost-effective, renewable, available in high quantity, biodegradable, have low fossil-fuel energy requirements and can offer good mechanical properties. Natural fibers are hygroscopic materials because of high content of water sorption sites (hydroxyl groups) and the deformation ability of the cell wall during water exposure. Thus, physical properties such as density, shape, stiffness, crystal structure of the fibers and therefore mechanical properties (tensile modulus and breaking stress) are impacted [3]. In the textile industry, the hygroscopic nature allows many applications such as: absorbing perspiration, transporting moisture and adjusting the relative humidity in the clothing microcosm.

Natural cellulosic fibers are obtained from various parts of the plants and generally classified based on the part of the plant from which they are extracted, such as seed, leaf and fruit. They are composed of various substances such as cellulose, lignin, hemicellulose and pectin. Cellulose, which is the primary reinforcing element of the cell wall, is made of linear chains of glucose residues aggregated into microfibrillar units [4]. These units possess a high crystalline content (inaccessible to water molecules) but also a paracrystalline component to which water molecules can gain access. Lignin is present in plant fibers in varying amounts; it is an amorphous crosslinked polymer composed of phenolic units. The cell wall also contains a hemicellulose and pectin component, which are predominantly amorphous polysaccharide [5]. Cotton and flax fibers are the most widely used natural fibers in various fields of application. Cotton fibers are a seed fibers which are considered as the purest form of cellulose, with around 90% cellulose content [6]. Flax fibers are a plant fiber which are composed of cellulose (80–90%), cellulosic fibrils embedded in hemicellulose (up to 7%), pectins (up to 5%) and proteins (0–1.5%). A few phenolics (1%), waxes and fats (0.5–1.0%), are also present [7]. Natural fibers can be modified using chemical treatment such as alkalization [8], mercerization, acylation, acetylation, peroxide treatment [9], salinization and benzoylation. Those treatments affect the contents of cellulose, hemicellulose and lignin within the fibers, yielding them more amorphous. Among those, cellulose-based regenerated fibers such as cellulose acetate fibers and viscose fibers have attracted attention due to their more ductile behavior which allow new specific applications [10]. Cellulose acetate fibers are a modified polysaccharide synthesized by the reaction of acetic anhydride with cotton linters or wood pulp [11]. In that respect, the hydroxyl groups are partially substituted by acetyl function, which reduces the number of primary sorption sites (which are generally assumed to be the OH groups) [12]. Viscose fibers are manufactured from cotton linters or wood pulp. First, cellulose is mercerized with a sodium hydroxide treatment, followed by xanthation substitution. Cellulose is then regenerated with a sulfuric acid treatment that converts cellulose into a more amorphous structure [13] leading to higher moisture content [14].

The determination of the water uptake at equilibrium of natural fibers by the gravimetric method at a given water activity invariably uses saturated salt solutions as a means of evaluating the water sorption properties of fibers [15]. In recent years, a dynamic vapor sorption (DVS) technique has been used to investigate the sorption and desorption of natural or regenerated cellulosic materials from a thermodynamic point of view [16]. This technique leads to reproducible data and can provide accurate isotherms for water activities up to 0.95. Recent studies have not allowed a perfect understanding of the water sorption behavior of cellulosic fibers. Two main factors can explain this issue: the complex internal geometry of the cell wall and the continuous nano-structural changes associated with the dynamic behavior of the cell wall macromolecular components. In order to understand the sorption mode and possible interactions between water molecules and cellulosic fibers, sorption models have been established [17]. These models describe water sorption isotherms of cellulosic materials; nevertheless, they are limited in the fitting of the experimental data for the whole range of water activities and for all types of cellulosic materials. This limitation on the fitting has been attributed by Labuza [18] to different mechanisms of water association with cellulosic fibers in different water activity regions. Depending on the nature of the fiber, some models are more or less relevant. The Ferro–Fontan model allows one to predict sorption isotherm in 90% of cellulosic products [19]. Peleg suggests a four parameters model, which can be used for both sigmoidal and non-sigmoidal isotherms [20]. The Smith model is convenient for sorption isotherms of biological materials, such as starch [21]. Chirife and Iglesias found that Halsey and Oswin models are also versatile for the description of polysaccharide systems [22]. The well-known GAB model based on multilayers and condensed systems is considered to be one of the most versatile models for water sorption in cellulosic materials (GAB model, Guggenheim–Anderson–de Boer) but failed to fit values obtained at high water activity (higher than 0.9) [23]. The Park model is more convenient to fit the water sorption isotherms in the whole range of water activity. This model corresponds to a multi-sorption mode, which can be dividing in three steps: (1) Langmuir sorption, (2) Henry’s law and (3) water clustering [24].

In this present work, a comparative study of water sorption and desorption of two natural cellulosic fibers, cotton and flax, and two regenerated cellulosic fibers, cellulose acetate and viscose, is presented and discussed. The obtained isotherms were modeled using the Park model in order to have information about the sorption mode and the interactions between water molecules and cellulosic fibers at different water activity. The obtained sorption properties results were correlated to the accessibility and the amount of sorption sites and also to the crystallinity level of the fibers. Tensile tests of the fibers were also performed at different water activities to determine the consequences on the mechanical properties. Water molecules were used as a probe to characterize the microstructure of the fibers in order to establish the relationship between the structure, the water sorption and the mechanical properties.

## 2. Materials and Methods

### 2.1. Materials

**Figure 1:**
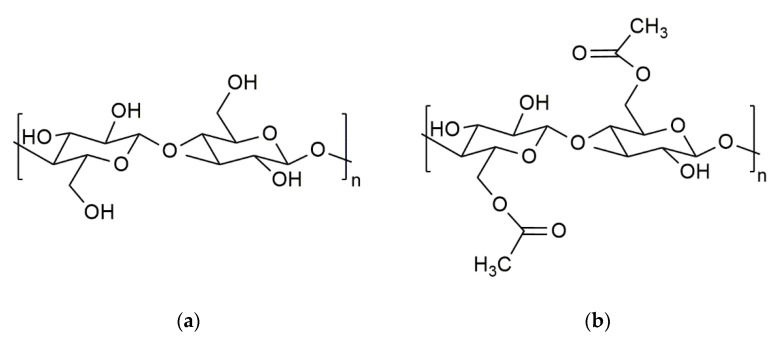
Chemical structure of: (**a**) cellulose; (**b**) cellulose acetate in cellulosic fibers.

Four cellulosic materials, cotton (C), flax (F), viscose staple (V) and cellulose acetate (CA) supplied by *Les Tissages de Charlieu* (Charlieu, France), were used in the experiments. Degree of substitution (DS) of CA was determined using the procedure described by Rodrigues et al. [25]. The characteristics of the fibers are given in Table 1, and the chemical structure of cellulose and CA are in Figure 1.

Different salts were used to equilibrate the fibers for mechanical characterization at defined water activities (*a_w_*). Potassium carbonate (K_2_CO_3_), ammonium nitrate (NH_4_NO_3_), sodium chloride (NaCl) and potassium nitrate (KNO_3_) were purchased from Sigma-Aldrich. All salts were A.C.S. grade. Distilled water obtained from a MilliQ™ purification system with a water resistivity greater than 18 MΩ cm was used to prepare the salt solutions.

### 2.2. Infrared Spectroscopy (FTIR)

FTIR spectra were recorded on a Nicolet iS10 infrared spectrometer from Thermo Fischer Scientific in attenuated total reflectance (ATR) mode with a diamond crystal. The scanning was conducted in the wave number range of 4000–400 cm^−1^ with 64 repetition scans for each sample. The resolution was set at 4 cm^−1^ during the measurement.

### 2.3. Wide-Angle X-ray Scattering (WAXS)

WAXS analyses in reflection (Bragg–Brentano) mode were carried out at room temperature using a Cu tube (λ=1.54 Å, 40 kV, 40 mA) and a nickel filter in order to remove the Kβ line and a Bruker D8 Advance diffractometer with a Bragg–Brentano configuration. The diffraction patterns were obtained in a 2θ range between 5° and 50° by a step of 0.02°. Fibers were deposited on corundum substrate with a thin transfer adhesive of low scattering response.

The crystallinity index ($X_{c}$) was determined by deconvolution method. $X_{c}$, which is defined as the ratio of the sum of area of cellulose crystalline peaks ($A_{cr}$) to the sum of the area of the total peaks of sample material, included the amorphous part ($A_{am}$) and the crystalline part, as defined by the following Equation (1):

(1) $$X_{c}\left(\%\right)=\frac{A_{cr}}{A_{cr}+A_{am}}\times 100$$

### 2.4. Vapor Water Sorption

Water sorption isotherms of the different fibers were determined at 25 °C by using the dynamic vapor sorption analyzer (DVS Advantage, London, United Kingdom). The vapor partial pressure was controlled by mixing dry and saturated nitrogen, using electronic mass flow controllers. The initial mass of the samples was between 15 to 40 mg. Each sample was pre-dried by exposure to dry nitrogen until the dry mass was obtained ($m_{0}$). A partial pressure of vapor (p) was then established within the apparatus and the mass of the sample ($m_{t}$) was followed as a function of time. The mass of the sample at equilibrium ($m_{eq}$) was considered to be reached when changes in mass with time (dm/dt) were lower than 2 × 10^–4^ mg min^−1^ for at least 10 consecutive minutes. Then, the samples were exposed to the following water activity (0.05, 0.10, 0.20, 0.30, 0.40, 0.50, 0.60, 0.70, 0.80, 0.90 and 0.95), before decreasing to 0 in the reverse order. The value of the mass gain at equilibrium (M) defined as $(m_{t}-m_{0})/m_{0}$ for each water activity ($a_{w}$) allowed for plotting the water sorption and desorption isotherms for each sample.

### 2.5. Mechanical Properties

Tensile properties of the fibers were obtained using a Shimadzu X+ testing machine equipped with a 100 N load cell. A gauge length of 10 cm and crosshead speed of 10 mm min^−1^ was used. Prior to mechanical tests, the fibers were equilibrated at different water activities: 0.47, 0.54, 0.66 and 0.87. Values of tensile modulus (*E*), stress at break (*σ*) and strain at break (*ε*) were determined from the stress–strain curve. The values of the tensile properties were the result of the arithmetic mean of the properties measured at least on seven different samples. Tensile tests were performed at 25 °C.

## 3. Analysis

The mathematical description of the sorption isotherms can bring useful information concerning the sorption mode and the interactions involved in the sorption process [17,30]. Several models have already been used to describe sigmoidal type isotherms. These models are classified into three different groups [22]: (i) empirical models (such as Smith, Oswin and Peleg models), (ii) semi-empirical models (such as Ferro–Fontan, Henderson and Halsey models) and (iii) models based on a multi-layer approach (Modified-Brunauer, Emmett, Teller (BET) model and GAB model) [31]. Park has proposed a model that offers a detailed description of sorption phenomena by expressing M versus $a_{w}$ [24]. This model comprises three terms, Equation (2):

(2) $$M=\frac{A_{L}b_{L}a_{w}}{1+b_{L}a_{w}}+k_{H}a_{w}+K_{a}a_{w}^{n}\;$$

The first term describes Langmuir sorption, which leads to a plateau of concentration when water activity increases, corresponding to the saturation of the specific sites of sorption. Langmuir’s terms, $A_{L}$, (Langmuir capacity constant) and $b_{L}$ (Langmuir affinity constant) have an influence in the first step of water sorption, at low water activity. The second term defines a linear evolution of the mass gain when the water activity increases (Henry’s law). Henry’s solubility coefficient, $k_{H}$, defines the slope of the isotherm in the second zone. The third term is a power function that represents the water aggregation phenomenon. The two last parameters, $K_{a}$, the equilibrium constant for the clustering reaction and n, the mean number of water molecules per cluster, can be linked to the equilibrium state corresponding to the aggregate formation in the last zone at high water activity:

$$zH_{2}O\overset{Ka}{\Leftrightarrow }\frac{z}{n}{\left(H_{2}O\right)}^{n}.$$

with z, the total number of water molecules sorbed. However, it should be noted that the n value represents a fitting parameter related to the mathematical calculation of the Park model and thus may be different from the real mean cluster size, which was determined at a different activity using the Zimm and Lundberg theory.

To evaluate the accuracy of the Park model to describe the experimental water sorption isotherms of the different fibers, the mean relative deviation (MRD) was used, and it is defined by the following Equation (3):

(3) $$MRD\left(\%\right)=\frac{100}{N}\overset{N}{\underset{i=1}{\sum }}\frac{\left|m_{i}-m_{pi}\right|}{m_{i}}$$

where $m_{i}$ is the experimental value, $m_{pi}$ is the predicted value, and N is the number of experimental data. The MRD is widely adopted throughout the literature, with a modulus value below 10% usually revealing a good fit [32]. The parameters of Park equation were determined by fitting using the software Origin 9.1.

As already pointed out, the high increase in uptake observed for BET type II isotherms at high activities is generally explained by a clustering phenomenon. Zimm and Lundberg have developed a method on the basis of statistical mechanisms, which analyzes this phenomenon from the single shape of the experimental isotherm [33,34].

They have developed a method that gives an interpretation of the solution thermodynamic behavior in geometric isotherms. Neglecting the isothermal compressibility of the polymer–permeant solution makes the free energy function of the system essentially dependent upon the first derivative of the activity with respect to the volume fraction. The elaborated relation appears as follows Equation (4):

(4) $$\frac{G_{s}}{V_{w}}=-\left(1-\Phi_{w}\right){\left[\frac{\delta \left(a_{w}/\Phi_{w}\right)}{\delta a_{w}}\right]}_{p,\;T}-1$$

where $G_{s}$ is the cluster integral, $V_{w}$ is the partial molecular volume of the permeant molecules, $\Phi_{w}$ is the volume fraction of the permeant molecules in the fibers.

A $G_{s}/V_{w}$ value equal to −1 indicates that water dissolves into polymer randomly, instead higher values, $G_{s}/V_{w}>1$ means that the concentration of water in the neighborhood of a given permeant molecule is greater than the average concentration of permeant molecules in the polymer. The quantity $G_{s}\Phi_{w}/V_{w}$ is the mean number of molecules in excess of the mean concentration of penetrant in the neighborhood of given permeant molecules. Thus, the mean cluster size (MCS) can be evaluated by the following Equation (5):

(5) $$MCS=1+\left[\frac{G_{s}\Phi_{w}}{V_{w}}\right]$$

MCS values have been calculated from Park parameters considering the following Equation (6):

(6) $$MCS=\frac{\rho_{w}/\rho_{p}{}^{2}}{M^{3}{\left(1+\frac{\rho_{w}/\rho_{p}}{M}\right)}^{2}}\times \left(k_{H}\cdot a_{w}+\frac{A_{L}\cdot b_{L}\cdot a_{w}}{{\left(1+b_{L}\cdot a_{w}\right)}^{2}}+K_{a}\cdot n\cdot a_{w}^{n}\right)$$

where $p_{w}$ is the water density and $p_{p}$ the density of the fiber, $A_{L},\;b_{L},\;k_{H},\;K_{a}$ and n are the Park parameters as explained above and M the mass gain at equilibrium.

## 4. Results and Discussion

### 4.1. Chemical Structure

**Figure 2:**
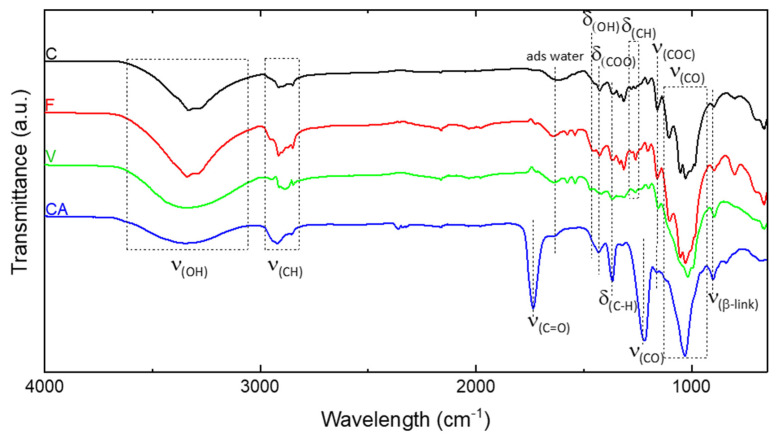
FT-IR spectrum of C, F, V and CA fibers.

The chemical composition of C, F, V and CA fibers was analyzed using FTIR-ATR. The interesting peaks are identified in Figure 2 C, V and F fibers have a similar chemical footprint [35,36,37]. The broad absorption band between 3600 and 3000 cm^−1^ is the characteristic of the O-H stretching vibration and hydrogen bond of the hydroxyl groups. The band at 1630 cm^−1^ was assigned to the bending mode of the adsorbed water. The peak at 1460 cm^−1^ corresponds to the OH bending and that at 1160 cm^−1^ is related to the C-O antisymmetric bridge stretching [38]. The absorption band at 1311 cm^−1^ was assigned to the CH_2_ stretching. In addition, the non-cellulosic polysaccharides were almost completely eliminated, as indicated by the absence of a peak at 1210 cm^−1^.

The spectrum of CA fibers provides strong evidence of acetylation by showing the presence of three important ester bonds at 1730 cm^−1^ (C=O ester), 1367 cm^−1^ (C-H bond in -O-CO-CH_3_ group) and the C-O stretching band of acetyl group at 1220 cm^−1^ [38]. The intensity of the broad band at 3400 cm^−1^ assigned to the stretching of the hydroxyl group decreased for CA compared with natural cellulose. A strong band at 1051 cm^−1^ was due to the C-O-C pyranose ring skeletal vibration. Another important aspect observed in the CA spectrum was the decreasing absorption intensity of the band located at around 3400 cm^−1^ assigned to the stretching of the hydroxyl group compared with C or F.

### 4.2. Crystalline Morphology

**Figure 3:**
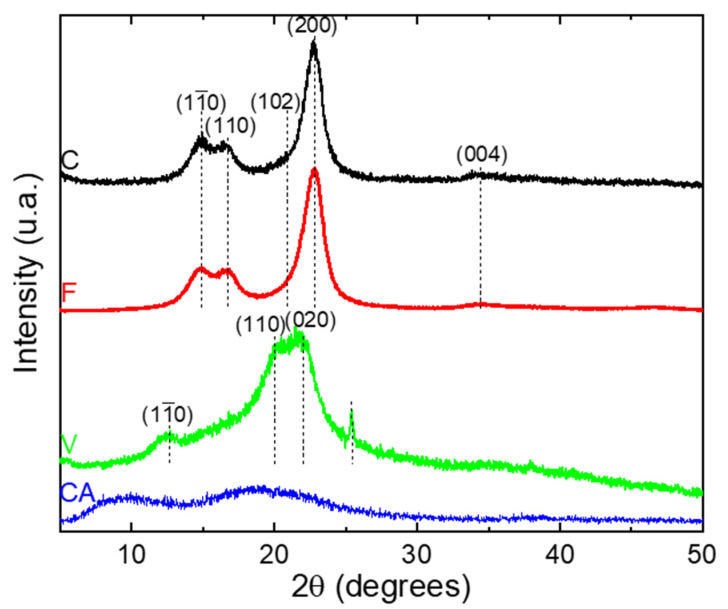
WAXS patterns of C, F, V and CA fibers.

The XRD analyses were performed in reflection mode. The obtained XRD patterns are presented in Figure 3. The diffraction diagrams of C and F fibers correspond to a typical XRD pattern of natural cellulose [39]. The diffraction peaks show five diffractions peaks at 2θ = 14.9°, 16.7°, 21.0°, 22.7° and 34.7° and were assigned to the diffraction planes ($1\bar{1}0$), (110), (102), (200) and (004) respectively [39]. These peaks belong to cellulose I_β_ crystalline structure.

The diffraction diagram of V fiber show three peaks at 2θ = 12.5°, 21.0° and 22.2° assigned to the ($1\bar{1}0$), (110), (020) planes, respectively [40,41,42,43,44], and were related to the cellulose II crystalline structure after mercerization [40]. An additional peak is observed at 2θ = 25.4° and was assigned to the anatase phase of titanium dioxide (TiO_2_), which gives a white coloration to the V fiber [44].

The XRD pattern of CA show a diffraction peak around 9.1° attributed to the crystalline peaks of CA II [45]. In addition, a diffraction peak at around 19.7° was commonly assigned to the less ordered or amorphous region of the cellulose chains [45].

A deconvolution procedure was applied to the XRD patterns according to the position of the different peaks defined previously, using the open software Fityk. The diffraction pattern can be decomposed into a broad amorphous halo centered at 2θ = 19.5° and Gaussian functions for each crystalline peak [46]. The curves resulting from the deconvolution are presented in the Supporting Information, Figure S1, and allow for the quantifying of the degree of crystallinity ($X_{c}$). Cellulosic fibers from various plants and different treatments differ considerably in their crystallinity index, as evidenced by a large number of investigative methods [47]. Consequently, $X_{c}$ values were calculated for each fiber and the data are listed in Table 2.

C and F fibers had a crystallinity index up to 60%, with 61 and 67%, respectively. The obtained values of $X_{c}$ were in good agreement to those obtained by Yueping et al. and Mikhalovska et al. [48,49]. The cellulosic derivatives display a low crystallinity index compared to the native cellulose ($X_{c}$ = 32% and less than 10% for V and CA, respectively). The decrease in crystallinity of CA has been explained by the fact that the substitution of the hydroxyl groups by acetyl with greater volume, break the inter- and intra-molecular hydrogen bonds of cellulose [38,50] and the decrease in crystallinity of V was attributed to the mercerization treatment as explained by Kafle et al. [40].

### 4.3. Water Sorption

#### 4.3.1. Sorption Isotherms

The hydrophilic behavior of cellulosic materials depends on their composition and their structural properties [3]. Cellulosic derivatives have hydroxyl groups on cellulose, hemicellulose and lignin, which are able to establish a hydrogen bond with water molecules. Moreover, a water vapor sorption occurs on non-crystalline areas and crystalline surfaces [51]. It has already been demonstrated that the crystallinity and the amount of hydrophilic sites could play a significant role on the water sorption behavior of fibers [52].

**Figure 4:**
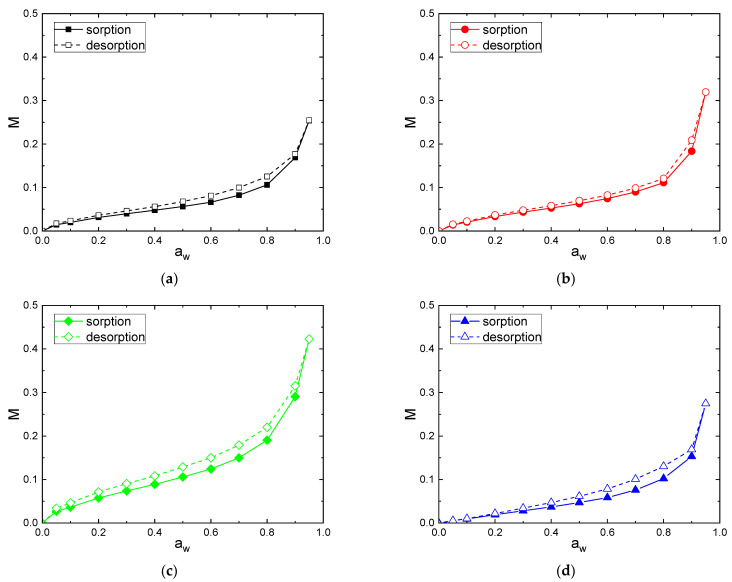
Water sorption and desorpton isotherms of: (**a**) C, (**b**) F, (**c**) V and (**d**) CA fibers.

Individual moisture sorption/desorption isotherms are shown in Figure 4 for the following fibers: C [Figure 4a], F [Figure 4b], V [Figure 4c] and CA [Figure 4d]. The sorption and desorption isotherm curves for all fibers have a sigmoid or S-shape, which corresponds to the type II in the classification of BET. This behavior is very typical of cellulosic-based materials [3,5] and can be generalized to many hydrophilic materials [53,54]. The sorption and desorption isotherms of CA fiber had a less pronounced sigmoid shape compared to the others fibers. This can be explained by a decrease in the amount of sorption sites, because hydroxyl groups are substituted by acetyl groups leading to a decrease in available sorption sites [55,56].

**Figure 5:**
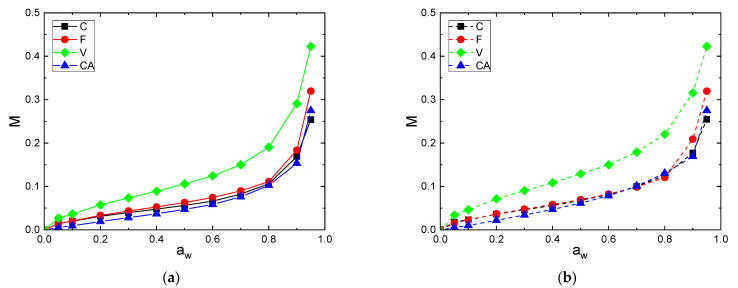
Water (**a**) sorption and (**b**) desorption of C, F, V and CA fibers.

The differences in sorption and desorption behavior are more clearly illustrated when the curves are overlaid in Figure 5a (sorption) and Figure 5b (desorption).

V exhibited higher water sorption and desorption capacities because of a large number of free hydroxyl groups presented in regenerative cellulose and a relatively low crystallinity index ($X_{c}$ = 32%). Then, F and C fibers had close water sorption and desorption uptakes in the whole range of water activity. Despite a higher crystallinity index, the F fiber had a slightly higher level of water uptake compared to the C fiber. This result is in agreement with that obtained by Mikhalovska et al. [49] and was explained by the presence of lignin in the F fiber, which was able to participate to the water sorption process. According to Hill et al., the reason why more highly lignified fibers show a higher water uptake may be related to the ability of the lignin network to accommodate water within the cell wall. The hydroxyl groups’ content to unit mass ratio is lower than with cellulose, thus OH accessibility would be higher in lignin compared with cellulose [5]. Cellulose acetate, due to the hydroxyl substitution into acetyl groups, exhibited the lowest water concentration despite its low crystallinity. Therefore, it appears that not only the crystallinity but also the swelling and sorption sites’ amount affected the water sorption capacity of cellulosic fibers.

To further understand the sorption and desorption mechanisms, Park’s model was used according to Equation (2). The Park’s model is consistent with a multistep sorption mode, usually observed for hydrophilic polymers, and can be divided into three terms. The Langmuir-type sorption relates to the sorption of the first water molecules on specific sorption sites within fibers. The Henry type law sorption describes the random dissolution of sorbed water molecules in the polymer for an intermediate water activity range. The third term corresponds to the water aggregation displayed by an exponential change in water mass gain. The values of the Park parameters for the all the fibers are summarized in Table 3. The examination of the fitting quality MRD, indicates that the Park’s model can describe the experimental sorption and desorption isotherms with a good accuracy.

**Figure 6:**
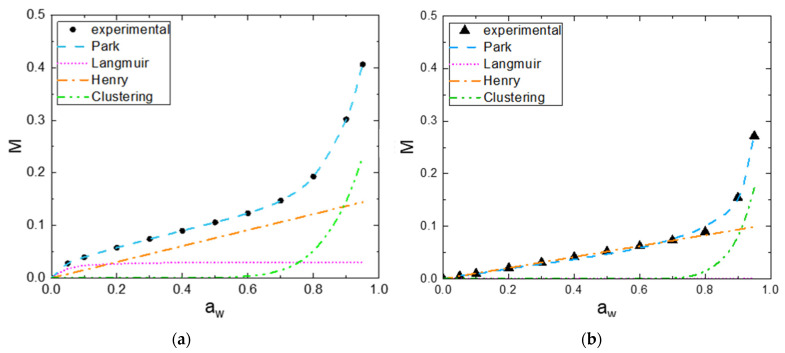
Experimental results and Park fit of experimental sorption isotherms for (**a**) V fiber and (**b**) CA fiber.

Two examples of the plotting of the isotherm sorption curve showing the three mode contributions are reported in Figure 6a,b for the V fiber and CA fiber, respectively.

Regarding the analysis of the Park model parameters, the chemical composition and the crystallinity index of cellulose have an influence on the three sorption modes. For the Langmuir mode, the evolutions of mass gain for all the fibers are shown in the Supporting Information, Figure S2a. The parameter $A_{L}$, which defines the Langmuir capacity, corresponds to the value at the plateau, whereas the Langmuir affinity constant $b_{L}$ governs the water activity where the plateau is reached. $A_{L}$ and $b_{L}$ values of CA are close to zero due to a low amount of Langmuir sorption sites and the presence of acetyl groups. F and C exhibit close values of $A_{L}$ and $b_{L}$, respectively, due to the same amount of hydroxyl groups in both fibers and the same water affinity for the specific groups. The activity to reach the plateau for these fibers was around 0.2 V exhibits the highest value $A_{L}$ because of the presence of a large accessible number of free hydroxyl groups presented in regenerative cellulose, and the $b_{L}$ value is lower compared to those obtained for F and C with a water activity to reach the plateau around 0.4.

**Figure 7:**
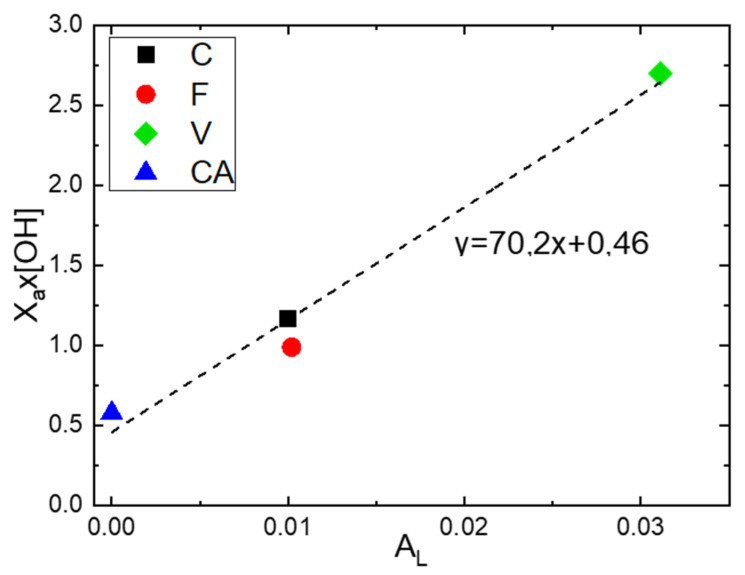
Evolution of the product $X_{a}\times \left[OH\right]$ as a function of $A_{L}$.

In order to evaluate the influence of the fraction of amorphous phase ($X_{a}$) and the number of hydroxyl groups per repeating unit of polymer [OH] on the Langmuir capacity constant $\;(A_{L}$), the product $X_{a}\times \left[OH\right]$ was calculated for each fiber and was represented as a function of $A_{L}$. (Figure 7). A linear increase in the product $X_{a}\times \left[OH\right]$ as a function of $A_{L}$ Was obtained. Thus, an increase in Langmuir sorption capacity results in an increase in the number of hydroxyl groups in the amorphous phase. However, the obtained value of $X_{a}\times \left[OH\right]$ for F was slightly below the straight line. This result can be explained by an overestimation of the value of $A_{L}$ due to the presence of lignin, which brings supplementary water sorption sites.

The evolutions of the mass gain of the Henry sorption mode as a function of the water activity for all fibers are shown in the Supporting Information, Figure S2b. The curves show a linear increase and the slope value corresponds to the Henry’s solubility coefficient $k_{H}$. The $k_{H}$ values exhibit an ascending sequence for C, CA, F and V, respectively. The $k_{H}$ parameter value defined as the random sorption of the water molecules in the fibers can be related to the level of amorphous phase, the presence of lignin and the amount of free volume of the fiber [57].

The third term corresponding to the water aggregation phenomenon, displayed by an exponential change in water mass gain as a function of the water activity, is observed in the Supporting Information, Figure S2c. The $K_{a}$ values (equilibrium constant for the clustering mechanism) and n (mean number of water molecules per cluster) can be linked to the equilibrium state corresponding to the formation of water molecules aggregating at high water activity. V fibers exhibit the highest water sorbed molecules in an aggregation state. For the other fibers, close values were obtained up to $a_{w}=0.9$ and differed at $a_{w}=0.95$ with a descending sequence for F, CA and C.

Park’s parameters were compared for the sorption and desorption isotherms for each fiber. Regardless of the fiber, differences were obtained only for the Henry sorption mode where a higher value of $k_{H}$ was obtained for the desorption isotherms. No significant differences were observed for $A_{L}$, $b_{L}$, $K_{a}$ and n, meaning that the Langmuir and water aggregation modes were not dependent on the sorption or desorption mode.

#### 4.3.2. Mean Cluster Size (MCS)

**Figure 8:**
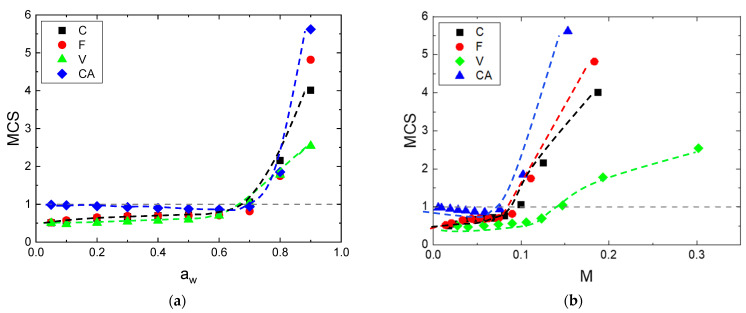
Evolution of MCS as a function of: (**a**) the water activity and (**b**) the water mass gain.

By using the theory of Zimm and Lundberg, it was possible to determine the MCS values from the parameters deduced from Park equation (Equation (3)). The plot of MCS versus the water activity is represented in Figure 8a. Regardless of the fibers, similar behavior was observed. MCS values are lower than unity at low water activity and then they increase at higher activities. This shape of curve corresponds to that currently observed from the BET II sorption isotherm [58]. The onset of water auto-association occurs at the same water activity, around $a_{w}=0.7$, in agreement with the results obtained from the third mode of the Park’s model (see in the Supporting Information, Figure S2c). At high water activity, typically for $a_{w}=0.95$, the MCS values exhibited an ascending sequence for V, F, C and CA with MCS= 2.5, 4, 5.5 and 7, respectively. This cluster size was in accordance with other literature data reported for F and C fibers [59]. MCS is also plotted as a function of the mass gain at equilibrium for the whole fibers, as shown in Figure 8b. Regardless of the fiber, the same shape of the curve was obtained, also showing two parts: a plateau with constant value of MCS, then an increase for the second part. As observed previously, the first part of the curve is due to water the molecules sorbed onto specific sorption sites and randomly distributed in the fiber. The second part is due to the interactions of a water molecule with another sorbed molecule when clusters are formed. The onset of clustering occurs in the mass gain range of 7–15%, with an ascending sequence for CA, F, C and V with *M* = 7.9, 9.4, 9.9 and 14.7%, respectively. This sequence of mass gain for water clusters’ formation starting goes in the opposite way of the MCS values. A higher MCS was obtained for a lower mass gain to begin water association. This result can be explained by the number of sorption sites available during the water sorption process [60]. CA had the lowest available sorption sites, which lead to a lowest mass gain to start the clusters’ formation and to the highest MCS values. The opposite effect was observed for V fiber.

### 4.4. Mechanical Properties

**Figure 9:**
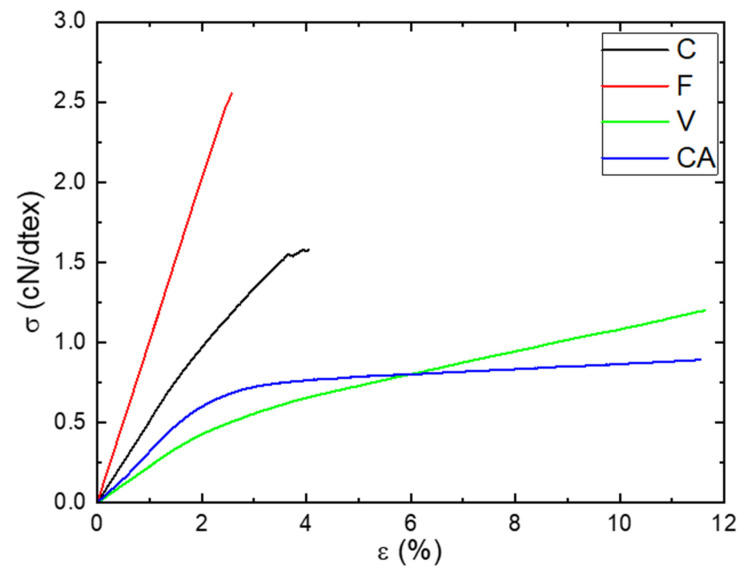
Evolution of the stress as a function the strain at *a_w_* = 0.47 and *T* = 25 °C for C, F, V and CA fibers.

The tensile behaviors of the fibers at *a_w_* = 0.47 and *T* = 25 °C were obtained from the stress–strain curves shown in Figure 9. Two shapes of the curves can be distinguished: one for the natural fibers (C and F) and the other one for the regenerated fibers (V and CA). The stress–strain curve of C showed two quasi-linear regions: the first one until 1.5% of deformation was attributed to the sliding of the microfibrils along with the progressive alignment with the fiber axis [61] and the second one was characteristic of an elastic behavior [62]. This behavior is very typical of natural fibers. The stress–strain curve of F showed a linear region until failure. This behavior was not in agreement with those obtained by Charlet et al. [62], where they demonstrated the presence of two linear regions as obtained for C. As explained by Placet et al. [63], this difference of behavior can be explained by the maturity and the sampling zone of the fiber. The stress–strain curves of the regenerated fibers showed the same shape: a first region until 2% of deformation corresponding to an elastic behavior and a second until failure corresponding to a plastic behavior. Values of tensile modulus (*E*), stress at break (*σ*) and strain at break (*ε*) are listed in Table 4.

F and C fibers had a higher Young modulus and stress at break but lower strain at break. Those differences can be explained to a large extent by the microstructure of the fibers. Natural fibers had a cellulose I_β_ crystalline structure associated to a higher crystalline index ($X_{c}$ > 60%), whereas regenerated fibers had a cellulose II crystalline structure associated to a lower crystalline index ($X_{c}$ < 35%). Moreover, concerning the ductile behavior of V and CA, it should be recalled that their fibers are made of continuous filaments, which certainly allows a higher deformability before the failure than for C and F, constituted of short fibers.

Tensile tests were also performed at different water activity to determine the influence of water molecules sorbed on the mechanical properties of the fibers.

**Figure 10:**
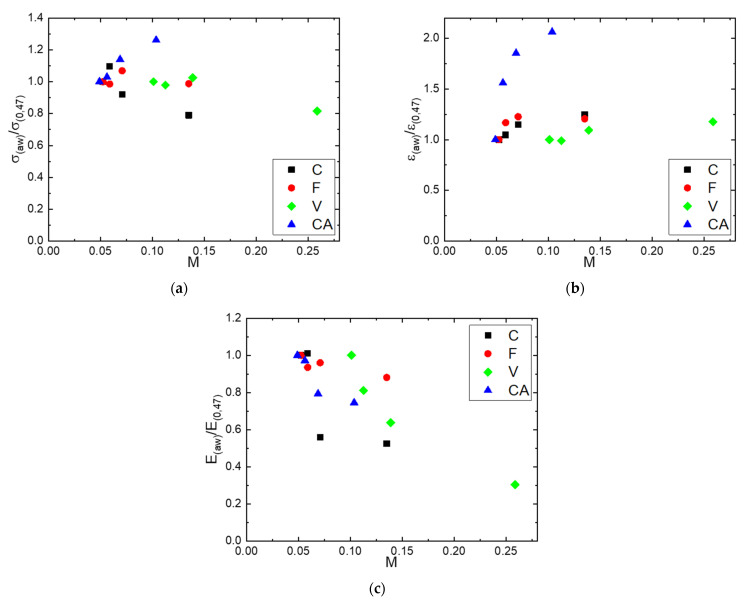
Evolutions as a function of the water mass gain of: (**a**) the normalized breaking stress ($\sigma_{a_{w}}/\sigma_{0.47})$, (**b**) normalized deformation at break ($\varepsilon_{a_{w}}/\varepsilon_{0.47})$ and (**c**) normalized module ($E_{a_{w}}/E_{0.47})$.

Figure 10a–c show the evolutions of the normalized breaking stress ($\sigma_{a_{w}}/\sigma_{0.47})$, normalized deformation at break ($\varepsilon_{a_{w}}/\varepsilon_{0.47})$ and normalized module ($E_{a_{w}}/E_{0.47}),$ respectively, as a function of the water mass gain. The normalized parameters were calculated considering the obtained values at a water activity equal to 0.47 (Table 3). The values of $\sigma_{a_{w}}/\sigma_{0.47}$ and $\varepsilon_{a_{w}}/\varepsilon_{0.47}$ were constant and equal to unity, indicating no dependency of the stress and strain at break on water molecules sorbed for C, F and V fibers. Nevertheless, for CA, the values of $\sigma_{a_{w}}/\sigma_{0.47}$ and $\varepsilon_{a_{w}}/\varepsilon_{0.47}$ increased as the mass gain increased. Again, the continuity of the constituting filaments may be a reason for that particularity. Concerning the tensile modulus, independently of the fiber nature, the same tendency was observed: $E_{a_{w}}/E_{0.47}$ decreased as the mass gain increased. It can be noted that the decrease in fiber rigidity depended on the water molecules sorbed by the fiber. A decrease of 70% was obtained for the F fiber, which had the higher water sorption capacity, whereas a decrease of only 25% was obtained for the CA fiber that had the lower water sorption capacity. This general behavior could be assigned to the plasticization phenomenon induced by the increase in the amount of sorbed water molecules as the water activity increased.

## 5. Conclusions

In conclusion, this study has demonstrated that there are considerable differences in the sorption/desorption behavior between cellulosic derivatives. Hydrophilic behavior of cellulosic materials depends on their composition and their structural properties. More specifically, it depends on the amount of water sorption sites (hydroxyl groups) and the amorphous fraction, because crystalline part is considered permeable. To understand the sorption mechanisms from the thermodynamic and kinetic point of view, a Park and PEK model were used, respectively.

The sorption and desorption isotherm curves for all fibers have a sigmoid or S-shape, which correspond to a type II classification of BET. The first part of the isotherm presents a concave form, which corresponds to the formation of the primary hydration sphere of the hydroxyl groups of cellulose. The linear evolution of the mass gain observed in the middle activity range was assigned to a random distribution of the water molecules. The convex part presented at high activity is attributed to the formation of water molecule clusters. V fibers exhibited the highest water sorption capacity allowed by the greatest hydroxyl groups’ accessibility and the large fraction of the amorphous phase, followed by flax and cotton, which present many hydroxyl sites but have a more crystalline structure. Cellulose acetate, due to the hydroxyl substitution into acetyl groups, exhibited the lowest water concentration despite its low crystallinity.

The Park model provides extremely good fits to the experimental data over the entire range of water activity for the four cellulosic fibers. This model is based on the concomitance of three sorption modes: Langmuir (sorption on specific sites), Henry (random sorption) and clustering (formation of aggregate). The Park’s model parameters were compared for the sorption and desorption isotherms for each fiber. Regardless of the fiber nature, differences were obtained only for the Henry sorption.

Results from tensile tests demonstrated that F and C fibers were more rigid, more resistant and less ductile than CA and V fibers due to a difference in the microstructure of the fibers. Regardless of the fiber nature, the presence of water sorbed molecules led to a decrease in tensile modulus due to the plasticization phenomenon. This decrease in tensile modulus was associated to the amount of water molecules sorbed by the fibers.

## Figures and Tables

**Table 1:** Mass percentage of the various constituents of various cellulosic and derivative fibers.

Material	Mass Composition (%)				Yarn Count (dtex)	DS	Ref.
	Cellulose	Hemicellulose	Pectin	Lignin			
C	82–99	3–6	0–5.7	-	100		[26,27,28,29]
F	64–85	10–20.6	2.3–12	0–5	400		
V	100	-	-	-	250	-	
CA	100	-	-	-	80	2.1 ^1^	[25]

^1^ obtained by titration according to previously published procedures [25].

**Table 2:** Crystallinity index of the fibers.

Material	$X_{c}\;(\%)$
C	61 ± 3
F	67 ± 2
V	32 ± 1
CA	<10

**Table 3:** Crystallinity index of the fibers.

**Material**		$A_{L}$	$b_{L}$	$k_{H}$	$K_{a}\;$	n	MRD (%)
C	S	0.0100	60	0.090	0.290	13	8.3
	D	0.0100	60	0.120	0.260	14	6.3
F	S	0.0102	81	0.107	0.516	18	2.6
	D	0.0106	70	0.126	0.504	18	3.2
V	S	0.0311	35	0.152	0.387	10	2.1
	D	0.0317	38	0.202	0.377	12	2.3
CA	S	0.0000	0	0.104	0.456	19	8.0
	D	0.0000	0	0.125	0.447	18	9.6

**Table 4:** Values of tensile module (*E*), stress at break (*σ*) and strain at break (*ε*) for *a_w_* = 0.47.

Material	E (cN/dtex)	σ (cN/dtex)	ε (%)
C	53 ± 3	1.6 ± 0.2	4.4 ± 0.4
F	120 ± 10	3.2 ± 0.6	2.7 ± 0.5
V	20 ± 2	1.1 ± 0.2	13.1 ± 1.8
CA	36 ± 3	0.9 ± 0.2	9.0 ± 1.6

## Data Availability

The data presented in this study are available as Supplementary Material.

## Supplementary Materials

The following supporting information can be downloaded at: https://www.mdpi.com/article/10.3390/polym14142836/s1, Figure S1: Deconvolution of X-ray diffraction curve of (a) C, (b) F, (c) V and (d) CA, Figure S2: Evolution of the sorption mass gain versus water activity in (a) Langmuir (b) Henry and (c) clustering modes for C, F, V and CA fibers.
