# Supplementary material for: Water Sorption and Mechanical Properties of Cellulosic Derivative Fibers
Source: Polymers (Basel). 2022 Jul 12;14(14):2836. doi: 10.3390/polym14142836 (PMC9322568; doi:10.3390/polym14142836)
Supplement: Supplementary file 1 [file polymers-14-02836-s001.zip › polymers-1804463-supplementary.pdf]

Supporting information

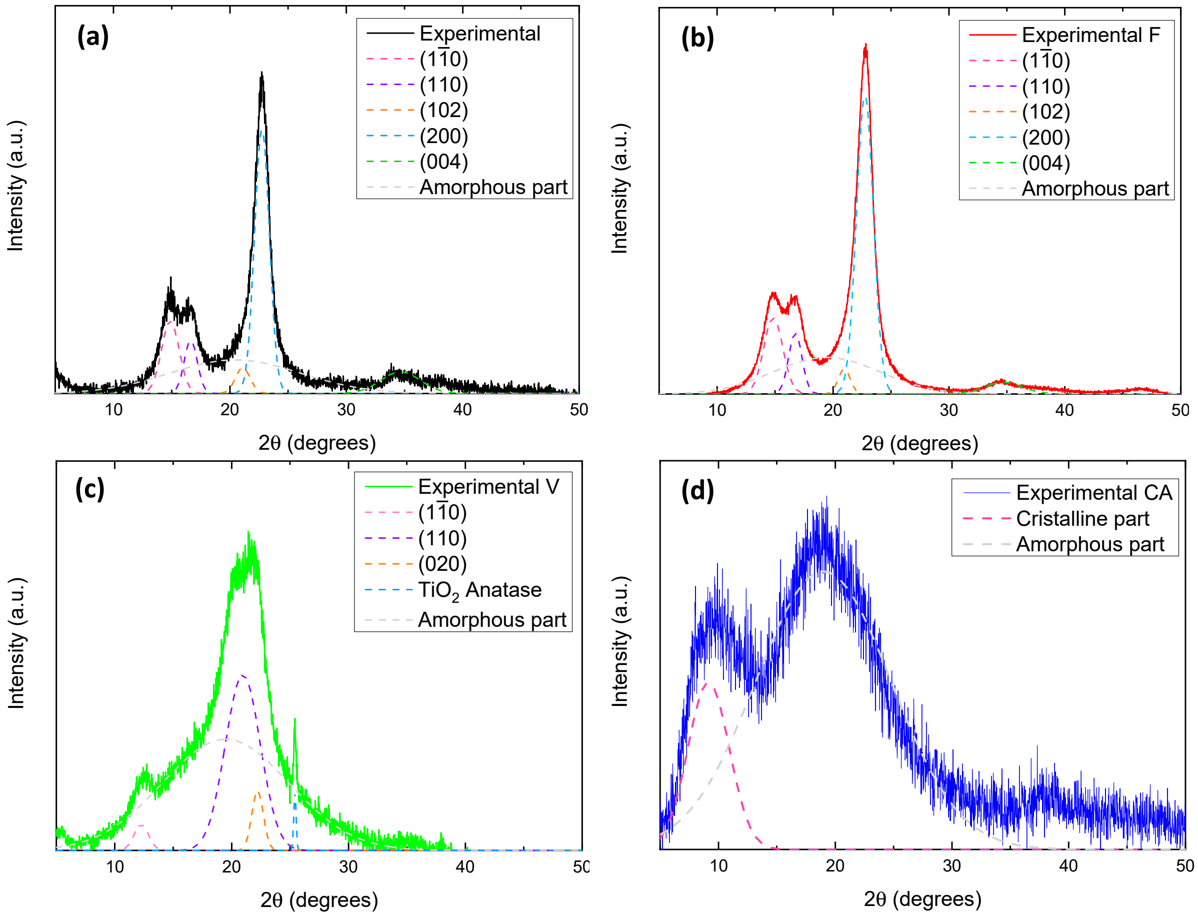

Figure S1. Deconvolution of X-ray diffraction curve of **(a)** C, **(b)** F, **(c)** V and **(d)** CA

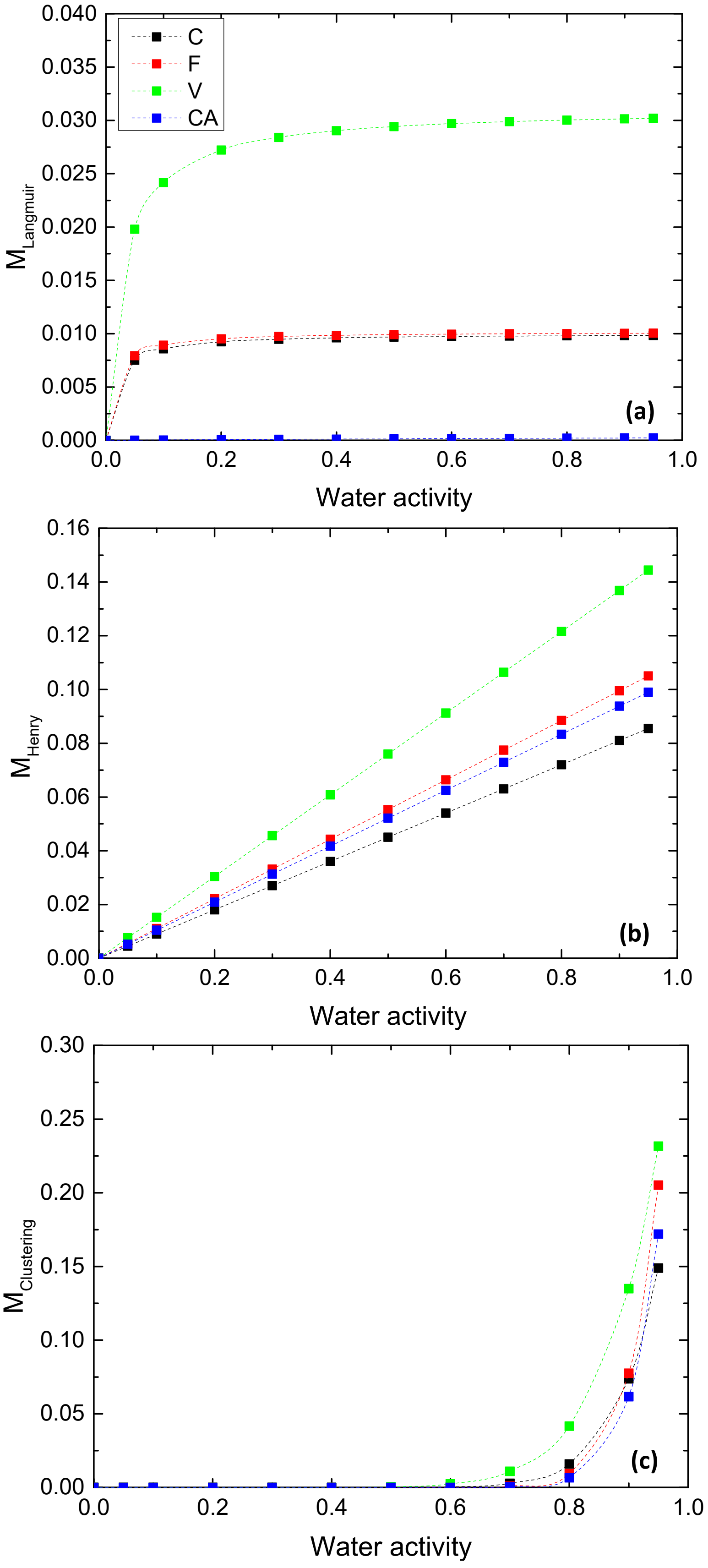

Figure S2. Evolution of the sorption mass gain versus water activity in **(a)** Langmuir **(b)** Henry and **(c)** clustering modes for C, F, V and CA fibers
